# Stimulation of Healing of Non-Infected Stagnated Diabetic Wounds by Copper Oxide-Impregnated Wound Dressings

**DOI:** 10.3390/medicina57101129

**Published:** 2021-10-19

**Authors:** Eyal Melamed, Alexei Rovitsky, Tohar Roth, Lior Assa, Gadi Borkow

**Affiliations:** 1 Department of Orthopaedics, Rambam Health Care Campus, Haifa 3109601, Israel; a_rovitsky@rambam.health.gov.il; 2 MedCu Technologies Ltd., Herzliya 4672200, Israel; tohar@medcu.com (T.R.); liorasa950@gmail.com (L.A.)

**Keywords:** copper oxide, diabetes, prospective study, wound dressings, wound healing

## Abstract

*Background and Objective*: Copper, a wide spectrum biocide, also plays a key role in angiogenesis and wound healing. Antibacterial wound dressings impregnated with copper oxide microparticles (COD) have been recently cleared by the U.S. FDA and other regulatory bodies for the treatment of acute and chronic wounds, including diabetic wounds. Our objective was to evaluate the capacity of COD in stimulating the healing of non-infected stagnated wounds in diabetic patients initially treated with standard of care (SOC) dressings. *Materials and Methods*: The trial was divided into the three following phases: 1–2 weeks of screening, during which the patients were treated with SOC dressings; 4 weeks of treatment, during which the COD was applied twice weekly; and 2 weeks of follow-up, during which the patients were again treated with SOC dressings. The wound conditions and sizes were assessed by clinical evaluation and a wound imaging artificial intelligence system. *Results*: Following 1 month of COD treatment, there was a clear reduction in the mean wound area (53.2%; *p* = 0.003), an increase in granulation tissue (43.37; *p* < 0.001), and a reduction in fibrins (47.8%; *p* = 0.002). In patients with non-weight-bearing wounds, the reduction in wound size was even more dramatic (66.9%; *p* < 0.001). *Conclusions*: The results of this study, showing a statistically significant influence of COD on wound healing of hard-to-heal wounds in diabetic patients, strongly supports the notion that copper oxide-impregnated dressings enhance wound healing directly. Further larger controlled studies should be conducted to substantiate our findings.

## 1. Introduction

The treatment of chronic wounds, such as diabetic foot wounds, often includes the use of wide-spectrum antimicrobial wound dressings [1]. Compounds such as honey, iodine, and silver have been incorporated into dressings to provide antimicrobial action. However, the clinical evidence demonstrating the superiority of the available antimicrobial dressings for improving the healing of leg wounds in diabetic individuals as compared to regular wound dressings is scarce, as analyzed by several systemic reviews of published trials (e.g., the Cochrane Database of Systematic Reviews (CDSR)) [2]. Furthermore, some studies reported no statistically significant differences in wound healing outcomes between antimicrobial and non-antimicrobial wound dressings [3,4]. 

Copper has been used in treating wounds for centuries by many different civilizations [5]. It has two key properties that endow it as an excellent active ingredient to be used in wound dressings. First, it is a potent wide-spectrum biocide, including against antibiotic-resistant bacteria and hard-to-kill bacterial spores and fungi [6,7]. Secondly, copper (a) stimulates angiogenesis [8], (b) induces dermal fibroblast proliferation [9], (c) upregulates collagen and elastin fiber components production by fibroblasts, (d) stimulates HSP-47, which is essential to collagen fibril formation [10], (e) serves as a cofactor of lysyl oxidase (LOX), which is needed for efficient extracellular matrix (ECM) protein cross-linking [11], and (f) stabilizes the skin ECM once formed [12]. 

Accordingly, the application of wound dressings containing copper oxide to non-infected wounds inflicted in genetically engineered diabetic mice resulted in increased gene and the in situ upregulation of pro-angiogenic factors (e.g., placental growth factor (PGF), hypoxia-inducible factor-1alpha (HIF-1α) and vascular endothelial growth factor (VEGF)), increased blood vessel formation, and enhanced wound healing as compared to control dressings (without copper) or wound dressings containing silver [13]. 

**Figure 1:**
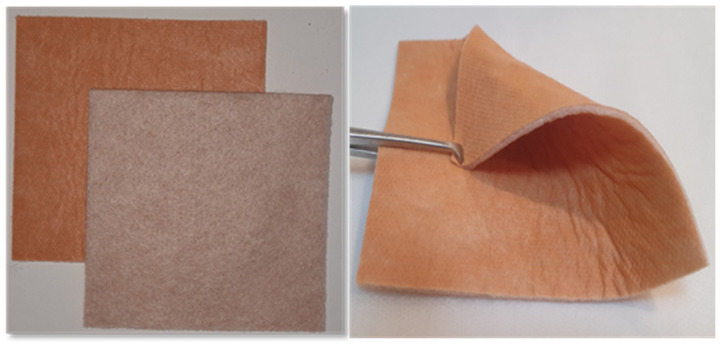
The studied copper oxide dressings. The antibacterial wound dressings with copper oxide (COD) are sterile wound dressings composed of an internal absorbent layer and one (**left panel**) or two (**right panel**) external nonwoven layers, with or without an adhesive contour. The external layer(s) are made of polypropylene nonwoven fabric, and the internal layer is made of a needle punch layer made of polyester and hydrofibers. The wound dressings achieve at least 800% weight/weight fluid absorbency. Both the internal and external layers are impregnated with copper oxide microparticles. The one or two external layers cover the internal layer from one or both sides, accordingly, and are applied directly to the wound.

Recently, antimicrobial wound dressings impregnated with cuprous oxide microparticles have been developed (COD, Figure 1) and cleared for use in acute and chronic wounds by the U.S. FDA and other regulatory bodies. In the current study, our aim was to evaluate the capacity of COD in treating non-infected foot wounds in diabetic patients in order to support our hypothesis that the application of COD may enhance wound healing of non-infected diabetic chronic wounds also.

## 2. Materials and Methods

Diabetic patients attending the outpatient clinic of Rambam Health Care Campus with non-infected chronic wounds between October 2019 and October 2020, at least 4 weeks from their last surgical intervention, were considered eligible for the study. Cooperative patients who met the inclusion criteria, without the exclusion criteria (Supplementary Table S1), and signed informed consent were enrolled in the study. 

**Figure 2:**
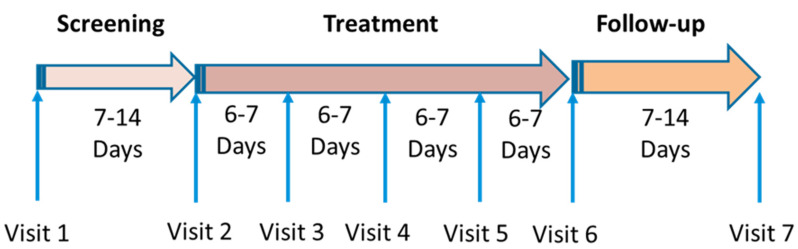
Study phases and visits to the clinic.

The trial was divided into three phases (Figure 2): the Screening Phase (1–2 weeks), the Treatment Phase (~1 month), and the Follow-up Phase (two weeks).

During the screening phase, patients were treated with SOC dressings. A reduction in the wound area of >35% was considered satisfactory healing and excluded the patients from further participation in the study. During the treatment phase, the COD was applied twice weekly. After 4 weeks of COD application, the treatment was changed to SOC, and the patients returned for a follow-up visit after 1–2 weeks. 

Wound area assessments were done by the wound imaging artificial intelligence system (Tissue Analytics; https://www.tissue-analytics.com/). The wound necrotic tissues, granulation tissues, fibrins, peri-wound redness, and potential wound infections were monitored by the examining physicians at each visit. All patients had a thorough physical examination done at the beginning and conclusion of the study. Blood tests for chemistry, blood count, liver and kidney function tests, and C reactive protein (CRP) were done at the beginning of the study and upon completion of the COD treatment phase. 

### Data Analysis

One-way repeated-measures ANOVAs were conducted to determine (1) the impact of the treatment with COD on the wound size/area over time (7 different time points—Visits 1–7), and (2) the difference in granulation tissue and fibrin over time during the Treatment Phase. Due to test assumptions, normality tests and equal variance tests were performed, while wound sizes were normalized to a percentage relative to the baseline wound size before the commencement of the Treatment Phase (Visit 2) [14]. Paired *t*-tests were performed as post-hoc tests between the time levels (consecutive visits) in order to investigate at which stages of treatment the decrease in wound size and increase in granulation and reduction in fibrin were significant. The differences in wound size reduction amongst non-plantar foot wounds and plantar wounds were examined by two-way repeated-measures ANOVA. Analyses were performed using SigmaPlot Version12.0 (Systat Software, San Jose, CA, USA).

## 3. Results

A total of 13 patients completed all three study phases and were included in the statistical analysis. All were neuropathic (Supplementary Table S2). 

Three of the patients also suffered from peripheral vascular disease (PVD) but had successful angiographic intervention prior to recruitment. The SOC treatments in the Screening Phase were hydrofiber dressings for eight patients and hydrofiber with silver for five patients. 

There were 3 plantar weight-bearing wounds (WBWs) and 10 non-weight-bearing wounds (NWBWs). The mean and standard deviation (SD) of the wound area at the beginning of the Treatment Phase was 9.26 ± 6.9 cm^2^ (range of 1.35–23.6 cm^2^). In the WBW subgroup and in the NWBW subgroup, the mean wound areas were 12.45 ± 9.7 cm^2^ (range of 1.35–16.76 cm^2^) and 8.31 ± 6.55 cm^2^ (range of 3.38–23.6 cm^2^), respectively.

**Figure 3:**
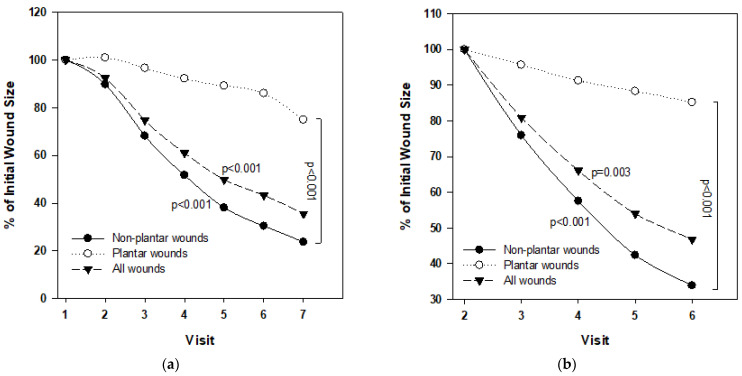
Repeated-measures ANOVAs of the mean wound size reduction and difference between the WBW and NWBW subgroups during the whole study (**a**) and during the Treatment Phase only (**b**).

**Figure 4:**
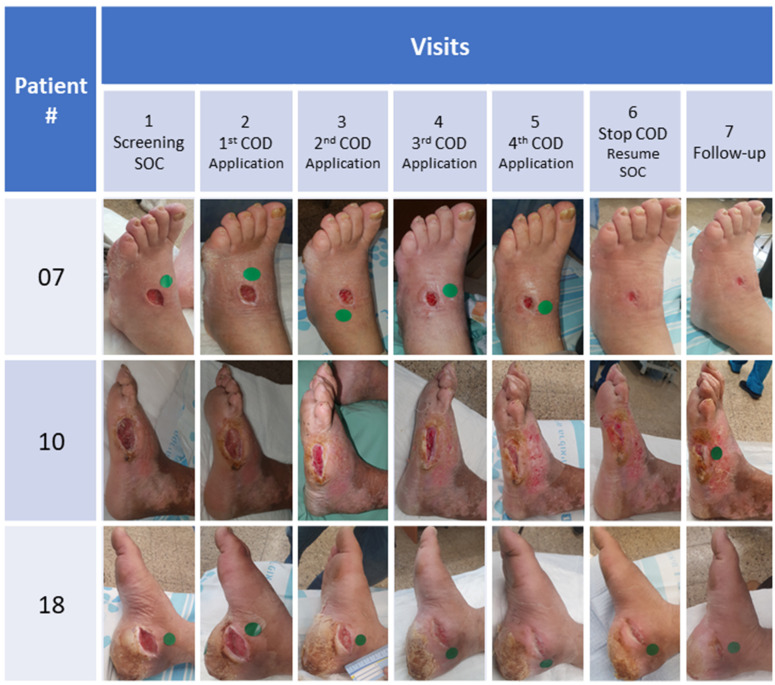
Pictures taken of the wounds throughout the study of 3 representative NWBW subgroups.

**Figure 5:**
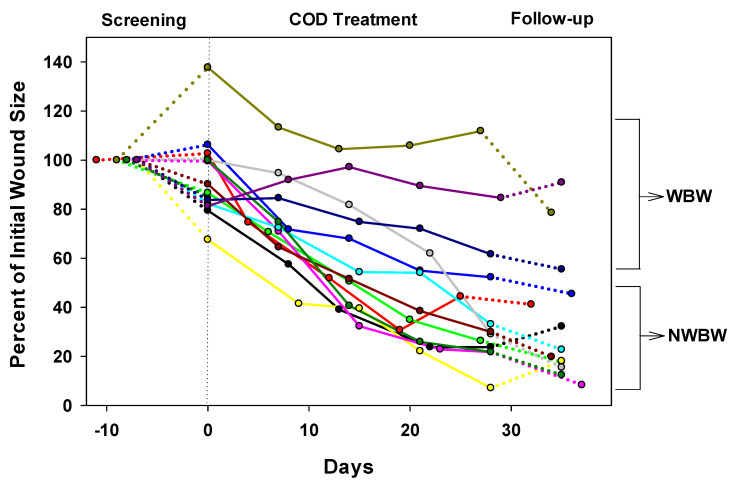
The sizes of the wounds normalized to the initial wound size per wound healing day for all 13 patients are shown. Day 0 represents the day the COD treatment started. Each color represents a different patient. The continuous line represents the Treatment Phase, which is preceded by the Screening Phase (dotted lines) and followed by the Follow-up Phase (dotted lines). The three upper lines belong to the three patients with weight-bearing wounds (WBW) and the other 10 lines belong to the 10 patients with non-weight-bearing wounds (NWBW). Each circle represents the date when the wound dressing was changed in the clinic and the wound size was measured by the wound imaging artificial intelligence system.

By the end of the Treatment Phase, there was a clear reduction of 65% (*p* < 0.001) in the mean wound area of all patients and of 76.8% (*p* < 0.001) in the NWBW subgroup (Figure 3a and Figure 4). The reduction in the NWBW subgroup varied between 48% and 93%. In the three WBW patients, the reduction varied between 0% and 26%. The individual lines representing the size of each patient per given day of treatment are shown in Figure 5. When analyzing the wound size reductions during only the Treatment Phase (Figure 3b), there were reductions of 53.2%, 66.9%, and 25% in the mean wound areas of all patients, and the NWBW and WBW subgroups, respectively (*p* = 0.003, *p* < 0.001, and NS, respectively). A two-way repeated-measures ANOVA between the patients with WBWs and those with NWBWs also revealed a statistically significant difference in the mean wound area reduction rates between these two groups of wounds (*p* < 0.001) when looking at the whole study, as well as when analyzing the Treatment Phase only (Figure 3a,b, respectively). 

**Figure 6:**
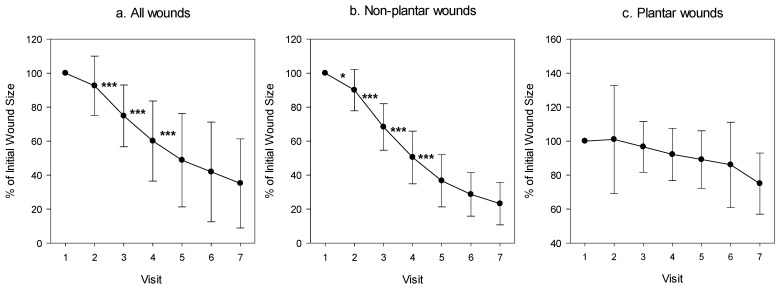
Percentage of wound area during the trial as compared to the wound size at the commencement of the study. The mean ± standard deviation of the wound area of (**a**) all patients, (**b**) of those suffering from a non-plantar foot wound, and (**c**) those suffering from a plantar wound. A paired *t*-test was conducted between each visit and the next one; * *p* < 0.05; *** *p* < 0.001.

As can be seen in Figure 6a, statistically significant reductions in the mean of the wound area occurred between each of the first three visits during the Treatment Phase in all 13 patients and in the NWBW patients (Figure 6b), but not in the WBW subgroup (Figure 6c). During the Follow-up Phase, the wound continued to decrease in size in 10 out of the 13 patients (Figure 4, Figure 5 and Figure 6).

**Figure 7:**
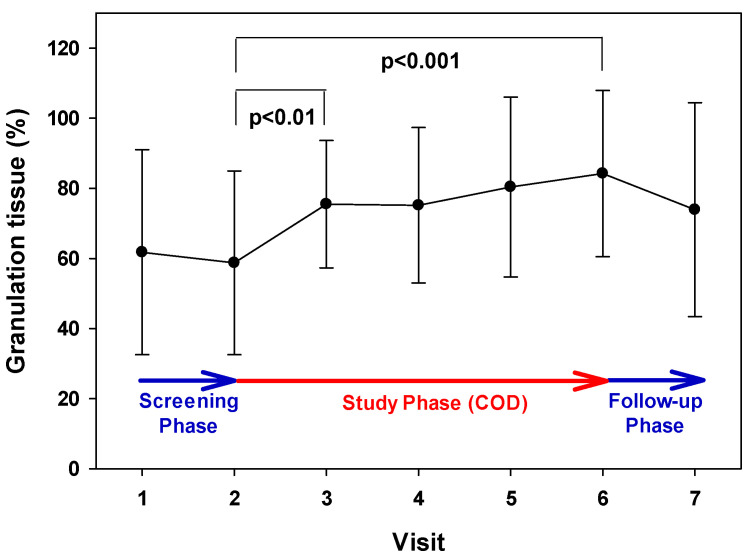
Increase in granulation tissue formation during the Treatment Phase. The mean and SD values of the mean of the granulation scores during each visit are presented. During the Treatment Phase (Visits 2–6), there was a statistically significant increase in the granulation tissue (43.37%; *p* < 0.001; one-way repeated measures ANOVA). Between Visit 2 and Visit 3, there was a statistically significant increase in the granulation tissue (28.5%; *p* < 0.01, paired *t*-test).

There was a gradual increase in the percent of granulation tissue following COD use (from 58.75% to 84.23%; a 43.37% increase; *p* < 0.001; one-way repeated measures ANOVA, Figure 7), already after one week of COD treatment (from 58.75% to 75.5%; a 28.5% increase; *p* < 0.01, paired *t*-test).

**Figure 8:**
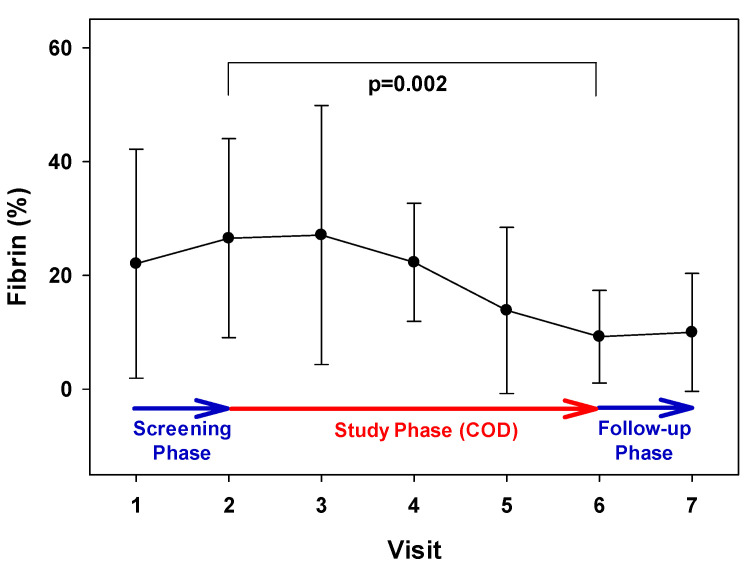
Reduction in fibrin tissue during the Treatment Phase. There was a statistically significant reduction in the percentage of fibrin during Visits 2 to 6 (47.8%; *p* = 0.002, one-way repeated measures ANOVA).

During the Treatment Phase, there was a reduction in the percent of fibrin tissue (from 26.53% to 13.85%; 47.8% decrease; *p* = 0.002, one-way repeated measures ANOVA, Figure 8).

Most wounds had less than 5% of necrotic tissue at the onset, and thus, no significant analysis could be performed regarding this parameter. There were also no significant changes in the amount of wound exudate discharge. None of the wounds had malodor.

There were no safety events related to COD use. No significant differences were noted in the blood parameters, kidney and liver functions, or in the overall physical medical conditions, before and after one month of the COD application. No infections in the wounds or peri-wound redness were observed.

## 4. Discussion

The current study is the first proof-of-concept clinical trial demonstrating the capacity of a simple application of wound dressings, COD, to improve wound healing of hard-to-heal wounds of diabetic foot patients. This is in contrast to the current standard of care of chronic stagnated wounds, which include the clearance of infection, if existent, followed by expensive, cumbersome, time-consuming, and painful treatment modalities, such as negative pressure wound therapy, pressure chamber treatments, and skin grafting.

Many factors, such as wound hypoxia and the inability to raise a sufficient inflammatory response, have been implicated as contributing to impaired wound healing, including in diabetic patients [15,16]. We have hypothesized that part of the incapacity of diabetic wounds to heal efficiently is due to low copper levels in the wound site [17], as copper is an essential element involved in all wound healing stages and processes [18,19]. Furthermore, we hypothesized that the slow in situ release of copper ions from wound dressings may not only confer protection to wounds from pathogens due to the intrinsic antimicrobial properties of copper [6,7] but, more importantly, may stimulate and enhance wound healing in hard-to-heal wounds [17]. 

This hypothesis was based on the fact that many factors involved in the wound healing processes are dependent on their interaction with copper, including VEGF expression [10,20], the export of fibroblast growth factor [21], the modulation of integrins expressed by supra-basally differentiated keratinocytes during the final healing phase [22], and the remodeling of the extracellular matrix proteins and their stabilization [23]. This hypothesis was strongly supported by the study conducted in engineered diabetic mice [13], in which the application of wound dressings containing copper oxide in comparison to control dressings (without copper) or commercial wound dressings containing silver to full-thickness non-infected wounds, inflicted in the mice backs, resulted in increased gene and in situ upregulation of pro-angiogenic factors (including PGF, HIF-1α, and VEGF), increased blood vessel formation, and enhanced wound closure. 

The results of the current study further support this hypothesis, especially in relation to the above-described mechanisms of action besides antimicrobial activity, as only diabetic patients with non-infected wounds were included in the study. Furthermore, the Screening Phase included only wounds that healed slowly or not at all with the use of SOC. Notably, we found that treating these non-infected, hard-to-heal wounds with COD improved all wound-healing parameters, including granulation tissue formation, epithelialization, and wound size reduction. The reduction in wound size was especially dramatic and statistically significant in the non-plantar wounds. This finding supports the well-accepted notion that pressure on a wound hinders wound healing regardless of any adjunctive treatment modality [24].

The positive effect of COD was noted in all patients with non-plantar wounds, including three suffering from PVD and three suffering from ischemic heart disease. Patients with significant renal insufficiency (creatinine > 3.0) were excluded from this study to prevent possible bias by extreme cases. However, we allowed patients with severe PVD to participate in the study if they had a successful angiographic intervention. These patients typically suffer from atherosclerosis of large and small arteries (patients with diabetes tend to obliterate the smaller sized arteries more); so, even after successful angiography, they should be regarded as ischemic wounds, which were evidenced by the failure to heal adequately in the screening phase with SOC dressings. The positive effect in lieu of ischemia can be explained by the positive effect that the copper ions released from the cuprous oxide microparticles had on the expression of HIF-1α, and other angiogenic stimulating factors. We noted a reduction in inflammation in post-op wounds, which were treated immediately after being operated on with the COD (unpublished results). It may be that the improvement in the wounds seen in the current study was also influenced by the anti-inflammatory properties of COD. We intend to explore this possibility further.

Improvement in wounds was noted in all wounds regardless of their initial baseline size and other interfering factors, other than weight-bearing pressure. We believe that triggering the healing response during COD treatment contributed to the continued healing in the Follow-up Phase. Our results are in accordance with 10 case reports we recently reported, in which COD not only conferred protection to the wound and the dressing from microbial contamination, and in some cases, helped to clear the wound infections, but in addition, stimulated skin regeneration and wound healing [25]. In the current study, all patients asked to continue being treated with the COD, and all non-plantar wounds closed within 1–15 weeks (average 7.5 ± 3.5 weeks) after the completion of the trial. 

There were no adverse events related to the COD, such as pain, irritation, odor or discomfort, kidney or liver malfunctions, or any effect on the general physical medical condition of the patients. This is in complete agreement with the high safety demonstrated in biocompatibility studies conducted with COD (e.g., [26]).

A limitation of the current study was the small proportion of patients that finished the trial. Another limitation of the study is that no stratification related to age or gender distribution was performed due to the small number of patients in each subgroup. In addition, the SOC dressings were not the same for all during the Screening Phase. The study was planned to convey minimal interference to the regular treatment regime. Although a statistically positive outcome of COD versus SOC was achieved, especially in the non-weight-bearing patients, further larger-scale randomized trials should be conducted for non-infected wounds in order to further substantiate the notion that COD not only confers antimicrobial protection to the wounds but actually stimulates wound healing.

## 5. Conclusions

The use of COD is a new concept in wound healing based on the ability of copper ions to endow antimicrobial activity as well as to stimulate angiogenesis and epithelialization. This first clinical trial with COD, showing a statistically significant influence of COD on healing (decreased wound size, increased granulation tissue, and decreased fibrin) is a proof-of-concept that COD can be beneficial in stimulating the direct healing of non-infected hard-to-heal wounds in diabetic foot patients.

## Supplementary Materials

The following are available online at https://www.mdpi.com/article/10.3390/medicina57101129/s1, Table S1: Inclusion and Exclusion Criteria, Table S2: Demographics and Wound Data at Baseline of Studied Patients.
