# Supplementary material for: Stimulation of Healing of Non-Infected Stagnated Diabetic Wounds by Copper Oxide-Impregnated Wound Dressings
Source: Medicina (Kaunas). 2021 Oct 19;57(10):1129. doi: 10.3390/medicina57101129 (PMC8538133; doi:10.3390/medicina57101129)
Supplement: Supplementary file 1 [file medicina-57-01129-s001.zip › medicina-1416276-supplementary.pdf]

Supplementary Table 1. Inclusion and Exclusion Criteria

Inclusion Criteria:

- Type 1 or type 2 Diabetes Mellitus
- Wound existed for at least 4 weeks
- No signs of sufficient healing despite SOC treatment
- Wound size of 2-30 cm^2
- The wound did not have signs of infection
    - If erythema was present at wound it was up to 0.5 mm from the wound edges
    - No thick-purulent nor purulent exudate was allowed
    - Green or black necrotic tissue constituted less than 20% of the wound area
- The wound did not have cavities or deep sinuses
- Having at least moderate blood perfusion to the affected limb as defined by either of the following:
    - palpable pulses
    - Ankle Brachial Index (ABI) > 0.6
    - if ABI > 1.3, then toe pressure of > 50 mmHg
    - Recent successful angiographic intervention
- Having a body mass index (BMI) of <40 Kg/m^2
- Having a glycosylated haemoglobin (HbA1c) of <10.0%
- Not systemic or topical antibiotic treatment in the week prior to enrollment in the study
- No wound deformity or bony projection that was severe enough to jeopardize wound healing as deemed to the treating physician

Exclusion Criteria:

- Active or unstable medical condition (cardiac, gastrointestinal, endocrine, neurological, liver, or kidney disease)
- Known of allergy to copper
- Active Charcot arthropathy
- Subjects with any of the following laboratory tests results:
    - Anemia (Hemoglobin < 8.5 g/dL)
    - Glycosylated hemoglobin (HbA1c) >10.0%
    - BMI > 40 Kg/m^2
    - White Blood Cells count > 11,000/μL
    - Platelets count < 100,000/μL
    - Liver function tests > 3 times upper normal lab values
    - Creatinine > 3 mg/dL
    - Albumin < 2.5 g/dL
- The wound size area has decreased by more than 25% per week or 35% in two weeks by SOC treatment during the screening phase prior to the commencement of COD
- Had visible bone exposure at wound site

Supplementary Table 2. Demographics and Wound Data at Baseline of Studied Patients

Number of patients	13
Age (years)	
Mean	58
Median	56.5
Standard Deviation	7.73
Range	46-71
Gender	
Males	8
Females	5
Main Background Diseases (#)	
Diabetes mellitus	13
Neuropathy	13
Ischemia heart disease	3
Peripheral vascular disease	3*
Retinopathy	2
Wound location	
Non-plantar foot wounds	10
Plantar foot wounds	3
Wound area (cm^2)	
Mean	9.26
Median	5.74
Standard Deviation	6.9
Range	1.35-23.6
Dressing type in the screening period (SOC)	
Hydrofiber	8
Hydrofiber with silver	5

*Successful angiography prior to treatment
